# Survival Outcomes in T3 Laryngeal Cancers: Primary Total Laryngectomy vs. Concurrent Chemoradiation or Radiation Therapy—A Meta-Analysis †

**DOI:** 10.3390/biomedicines11082128

**Published:** 2023-07-28

**Authors:** Karthik Nagaraja Rao, Prathamesh S. Pai, Prajwal Dange, Luiz P. Kowalski, Primož Strojan, Antti A. Mäkitie, Orlando Guntinas-Lichius, K. Thomas Robbins, Juan P. Rodrigo, Avraham Eisbruch, Robert P. Takes, Remco de Bree, Andrés Coca-Pelaz, Cesare Piazza, Carlos Chiesa-Estomba, Fernando López, Nabil F. Saba, Alessandra Rinaldo, Alfio Ferlito

**Affiliations:** 1 Department of Head and Neck Oncology, All India Institute of Medical Sciences, Raipur 492099, India; prajwal.dange@gmail.com; 2 Department of Head Neck Surgery, Tata Memorial Hospital, Mumbai 400012, India; drpspai@gmail.com; 3 Department of Head and Neck Surgery and Otorhinolaringology, A.C. Camargo Cancer Center, São Paulo 01509, Brazil; lp_kowalski@uol.com.br; 4 Department of Radiation Oncology, Institute of Oncology Ljubljana, Faculty of Medicine, SI-10000 Ljubljana, Slovenia; pstrojan@onko-i.si; 5 Research Program in Systems Oncology, Department of Otorhinolaryngology, Head and Neck Surgery, Faculty of Medicine, Helsinki University Hospital, University of Helsinki, 00014 Helsinki, Finland; antti.makitie@hus.fi; 6 Department of Otorhinolaryngology, Jena University Hospital, 07747 Jena, Germany; orlando.guntinas@med.uni-jena.de; 7 Department of Otolaryngology Head and Neck Surgery, Southern Illinois University, Carbondale, IL 62901, USA; kthomasrobbins@gmail.com; 8 Department of Otolaryngology, Hospital Universitario Central de Asturias-Instituto de Salud del Principado de Asturias (ISPA), 33011 Oviedo, Spain; jprodrigo@uniovi.es (J.P.R.); cocaandres@uniovi.es (A.C.-P.); lopezafernando@uniovi.es (F.L.); 9 IUOPA, University of Oviedo, 33006 Oviedo, Spain; 10 CIBERONC, Instituto de Salud Carlos III, 28029 Madrid, Spain; 11 Department of Radiation Oncology, University of Michigan Medicine, Ann Arbor, MI 48109, USA; eisbruch@med.umich.edu; 12 Department of Otorhinolaryngology and Head and Neck Surgery, Radboud University Medical Center, 6525 GA Nijmegen, The Netherlands; robert.takes@radboudumc.nl; 13 Department of Head and Neck Surgical Oncology, University Medical Center Utrecht, Heidelberglaan 100, 3584 CX Utrecht, The Netherlands; r.debree@umcutrecht.nl; 14 Otorhinolaryngology—Head and Neck Surgery, ASST Spedali Civili di Brescia, School of Medicine, University of Brescia, 25121 Brescia, Italy; ceceplaza@libero.it; 15 Otorhinolaryngology—Head & Neck Surgery, Donostia University Hospital, 20014 Donostia, Spain; chiesaestomba86@gmail.com; 16 Department of Hematology and Medical Oncology, The Winship Cancer Institute, Emory University, Atlanta, GA 30322, USA; nfsaba@emory.edu; 17 ENT Unit, Policlinico Città di Udine, 33100 Udine, Italy; dottalerinaldo@gmail.com; 18 Coordinator of the International Head and Neck Scientific Group, 35100 Padua, Italy; profalfioferlito@gmail.com

**Keywords:** laryngeal cancer, T3, organ preservation, head and neck cancer, total laryngectomy

## Abstract

Background: The management of cT3 laryngeal cancers remains controversial, with studies recommending surgical or non-surgical approaches. Despite the many papers that have been published on the subject, there is a lack of studies showing which treatment has better results in terms of survival. Objective: To determine the difference in survival outcomes following total laryngectomy (TL), concurrent chemoradiation (CRT) or radiation therapy (RT) alone in T3 laryngeal cancers. Methods: Search of PubMed, Scopus, and Google Scholar databases from 1995 to 2023 employing specific keywords and Boolean operators to retrieve relevant articles. Statistical analysis was conducted using a random-effects model, and heterogeneity was evaluated using the Q-test and I^2^ statistic. Funnel plot asymmetry was assessed using rank correlation and regression tests. Results: The qualitative data synthesis comprised 10,940 patients from 16 included studies. TL was performed in 2149 (19.4%), CRT in 6723 (61.5%), RT in 295 (2.7%), while non-surgical treatment was not specified in 1773 (16.2%) patients. The pooled 2-year overall survival (OS) rates were TL = 73%, CRT = 74.7%, RT = 57.9%, 3-year OS rates were TL = 64.3%, CRT = 62.9%, RT = 52.4%, and 5-year OS rates were TL = 54.2%, CRT = 52.7%, RT = 40.8%. There was a significant heterogeneity in the included studies. There was no statistically significant difference in 2-year OS (logOR= −0.88 (95% confidence interval (CI): −1.99 to 0.23), *p* = 0.12), 3-year OS (logOR = −0.6 (95% CI: −1.34 to 0.15), *p* = 0.11), and 5-year OS (logOR = −0.54 (95% CI: −1.29 to 0.21), *p* = 0.16) between TL and CRT. Instead, there was significant difference in 2-year OS (logOR= −1.2383 (95% CI: −2.1679 to −0.3087), *p* = 0.009), 3-year OS (−1.1262 (95% CI: −1.6166 to −0.6358), *p* < 0.001), and 5-year OS (−0.99 (95% CI: −1.44 to −0.53)), *p* < 0.001) between TL and RT alone. Conclusions and Significance: TL followed with adjuvant (chemo)radiation on indication and CRT with salvage surgery in reserve appear to have similar OS outcomes. Both resulted in better OS outcomes compared to RT alone in the treatment of T3 laryngeal cancers. If patients are unfit for chemotherapy, making CRT impossible, surgery may become the choice of treatment.

## 1. Introduction

Laryngeal cancers account for 20% of all head and neck squamous cell carcinomas [1]. Over the past three decades, treatment of these cancers has undergone a significant evolution. While total laryngectomy (TL) has long been regarded as the gold standard for treating advanced laryngeal cancers, the landscape has shifted in the past two decades due to well-conducted randomised controlled trials (RCTs) [2,3]. These trials compared surgical approaches with non-surgical treatments, leading to a paradigm shift in overall therapeutic orientation. The primary endpoint of these RCTs was not just to assess survival advantage of one treatment over the other, but also to assess toxicity, chances for larynx preservation, and overall quality of life. Therefore, despite these well-controlled RCTs, there is still debate on the real survival improvement according to the different therapeutic approaches employed for advanced laryngeal cancers, especially considering the T3 category. The success of laryngeal preservation with CRT and RT depends on various factors, including age, coexisting medical conditions, tumour volume, extent of primary and nodal disease, access to post-treatment rehabilitation and support, and the overall experience of diagnostic and treatment teams [4].

Laryngeal (and also hypopharyngeal) cancers are one of the rare exceptions in oncology, as their survival figures have not improved over the past decades [5,6]. This stagnation in survival progress is a compelling reason for further studies. It is important to note that the survival of TL and non-surgical organ reservation protocols (CRT and RT) in T3 laryngeal tumours have not been systematically analysed. A growing body of evidence suggests that the increased utilisation of non-surgical laryngeal preservation strategies, instead of opting for TL, in managing locally advanced disease, might have decreased survival rates among patients with laryngeal cancer [7,8].

The management of cT3 laryngeal tumours (an advanced neoplastic lesion, but still limited within the anatomical boundaries of the larynx) remains controversial, with several studies advocating for surgical or non-surgical protocols [9]. Our study is the first comprehensive meta-analysis specifically focusing on the survival outcomes of TL compared with those of CRT or RT alone exclusively for treatment of T3 laryngeal cancers.

## 2. Methodology

### 2.1. Reporting and Registration

This study adheres to the guidelines outlined by PRISMA (Preferred Reporting Items for Systematic Reviews and Meta-Analyses) to ensure comprehensive reporting [10]. The meta-analysis was appraised by AMSTAR and was determined to be of high quality. The PROSPERO registration number is CRD42023439285.

### 2.2. Search Strategy

Our study followed the AMSTAR (Assessing the Methodological Quality of Systematic Reviews) guidelines [11], incorporating PubMed, Scopus, and Google Scholar databases for comprehensive coverage of the published literature. Only English-language papers from 1995 to 2023 were included.

### 2.3. Search Syntax

Employing Boolean operators (NOT, AND, OR) and the following keywords: “T3”, “Larynx”, “Carcinoma”, “Cancer”, “Total laryngectomy”, “Organ preservation” “Comparison”, “Radiation therapy”, “Chemoradiotherapy”, “Surgery”, “Survival”, and “Outcomes” we retrieved the results. The data retrieval was completed on 21 June 2023.

### 2.4. Data Screening and Selection

**Figure 1:**
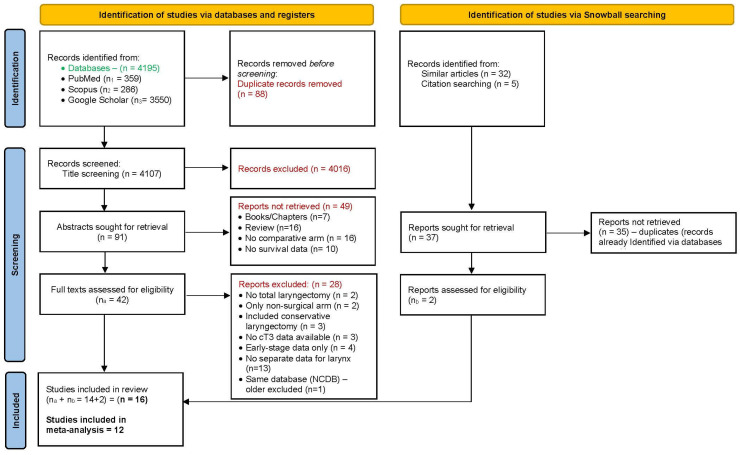
PRISMA flow diagram.

Two investigators (KNR and PD), screened the retrieved articles initially, considering factors such as article type, title, and abstract. The eligible reports were then compiled, and an extensive full-text analysis was performed, including examining references in relevant articles using snowball searching (Figure 1). The senior author (PSP) resolved any disagreements regarding inclusion of papers.

### 2.5. Inclusion and Exclusion Criteria

A meta-analysis was conducted on studies involving treatment-naïve cT3 non-metastatic laryngeal cancers, comparing survival outcomes of the surgical and non-surgical treatment strategies (CRT or RT alone). The surgical treatment approach considered was primary TL with adjuvant therapy based on post-operative histopathology. Non-surgical organ preservation strategies included RT alone or CRT with or without prior neoadjuvant chemotherapy (NACT). Only original articles published in peer-reviewed journals were included in the analysis. The selected papers were required to report at least one outcome following surgery, such as overall (OS), disease-free (DFS), or disease-specific survival (DSS) rates. Exclusion criteria were non-human studies, salvage laryngectomy, any form of partial laryngectomy, recurrent, second primary or metastatic tumours, studies without reported operative outcomes, absence of data for cT3 laryngeal subset of tumours, studies lacking a comparative arm. In addition, review articles, meeting abstracts, case reports, editorial letters, and other forms of publication as well as studies with incomplete data or insufficient information were also excluded from the meta-analysis.

### 2.6. Data Extraction

Two authors (KNR and PD) independently conducted the screening of all included articles. The study characteristics, including author, year of publication, country, sample size, type of study, level of evidence, Newcastle–Ottawa Scale (NOS) score, risk of bias assessment, number of cT3, number of T3 treated with TL, number of T3 treated with CRT, RT alone and non-surgical organ preservation (RT only or CRT—not specified), were meticulously recorded. In addition, the survival data, including 2-, 3-, and 5-year OS, DFS, and DSS were extracted from the respective published texts together with the respective Kaplan–Meier survival graphs. The subset data for cT3 laryngeal subsites were not available as these cases were clubbed together as cT3 in most of the included articles. The details of the chemotherapy regime were also not available in the majority of the studies. All the gathered information was then compiled and organised in a table format (Table 1). 

### 2.7. Quality Assessment

#### 2.7.1. Level of Evidence

Two authors (KNR and PD) independently assessed the level of evidence for the eligible studies, following the criteria outlined by the Oxford Centre for Evidence-Based Medicine (OCEBM) [28]. 

#### 2.7.2. Evidence Quality

The authors assessed the methodological quality of the included studies. The quality evaluation was performed using the Newcastle–Ottawa Scale, which assigns a score ranging from 0 to 9 based on specific criteria [29]. Articles with a score greater than five were selected for the systematic review, indicating better methodological quality.

#### 2.7.3. Risk of Bias Assessment

The risk of bias assessment tool, ROBINS-I, recommended by the Cochrane Handbook for non-randomised studies, was employed to determine the bias systematically [30]. The assessment encompassed seven domains: patient selection, confounding variables, classification of intervention, deviation from intended intervention, measurement of outcomes, incomplete data, and selective reporting. Using RevMan v.5.4 (Cochrane Collaboration in Copenhagen, Denmark), the studies were graded as low risk, unclear risk, or high risk based on their bias potential (Supplement Figures S1 and S2).

#### 2.7.4. Statistical Analysis

The analysis utilised the log odds ratio as the outcome measure, and a random-effects model was applied to the data. The amount of heterogeneity, estimated by the DerSimonian–Laird estimator [31], was denoted as tau^2^. The analysis also reported the Q-test for heterogeneity [32] and the I^2^ statistic. An I^2^ > 50% and *p* < 0.1 indicated significant heterogeneity [33]. A prediction interval for the actual outcomes was provided if any heterogeneity was detected (i.e., tau^2^ > 0, irrespective of the Q-test results). Studentized residuals and Cook’s distances were examined within the model to identify potential outliers and influential studies. Studies with studentized residuals exceeding the 100 × (1 − 0.05/(2 × *k*th)) percentile of a standard normal distribution were considered potential outliers (employing a Bonferroni correction with two-sided alpha = 0.05 for k studies in the meta-analysis) [34]. Additionally, studies with Cook’s distances greater than the median plus six times the interquartile range of Cook’s distances were deemed influential [35]. Funnel plot asymmetry was assessed using rank correlation and regression tests, employing the standard error of observed outcomes as a predictor [36]. A *p*-value < 0.05 was considered statistically significant. The calculations were performed using Jamovi software for Windows version 2.3.28, built on the R statistical package [37,38].

## 3. Results

### 3.1. Literature Retrieval and Data Extraction

The initial literature search, using specific search terms, yielded 4195 references. After removing 88 duplicates, 4107 unique articles were left for analysis. Subsequently, upon screening the titles, we excluded 4016 references that did not align with the objectives of our study. From the remaining 91 texts, we further excluded 49 based on specific criteria: 7 were book chapters, 16 were review articles, 16 lacked a comparative arm, and 10 had no survival data available. Following a thorough analysis of the full texts of the remaining 42 references, we rejected 28 for various reasons. Two studies involved surgeries other than TL, two studies included the results from surgical arm only, three studies focused solely on conservative laryngectomy, three studies lacked available cT3 data for analysis, four studies provided data only for early-stage cancers, thirteen studies merged the T3 laryngeal and hypopharyngeal sites, and two studies were derived from the same database (the older was excluded to ensure there was no duplication of results). To broaden our search, we conducted an additional snowball search based on similar articles and citation searching, resulting in 37 additional articles. However, upon review, 35 of these articles were identified as duplicates already found through the initial database search. The remaining two articles met the eligibility criteria and were included in the meta-analysis, bringing the total number of articles to sixteen [12,13,14,15,16,17,18,19,20,21,22,23,24,25,26,27] (including the two additional articles and the fourteen from the database search). Among these sixteen articles, six had provided data for both CRT and RT alone: two out of six articles had provided separate data for TL, CRT, and RT alone [18,21], while in four out of six articles, separate data for CRT and RT alone were not available and were therefore classified as “non-surgical organ preservation method not specified” [19,22,24,27]. Finally, seven articles had provided separate data for TL and CRT [16,18,20,21,23,25,26], seven articles for TL and RT alone [12,13,14,15,17,18,21], and in four articles, TL was compared with unspecified non-surgical methods of organ preservation [19,22,24,27] (Figure 1). Unfortunately, the information on tumour extension inside the larynx and the reasons for T3 classification were not available for subset analyses. We extracted the necessary data from published Kaplan–Meier graphs for qualitative data synthesis and meta-analysis.

### 3.2. Quality of Included Studies

A summary of the main characteristics observed in the included studies is presented in Table 1. The qualitative data synthesis involved 10,940 cases from 16 articles [12,13,14,15,16,17,18,19,20,21,22,23,24,25,26,27] with cT3 laryngeal cancer. TL was performed in 2149 (19.4%), CRT was the choice of organ preservation in 6723 (61.5%) cases, RT only was provided in 295 (2.7%) cases, and in 1773 (16.2%) cases, the type of non-surgical organ preservation method was not specified (CRT or RT alone). All included studies were retrospective comparative cohort studies and classified as OCEBM level III evidence. These studies originated from 11 countries, including the USA (*n* = 6) [16,17,20,25,26,27], and one article each from Brazil [13], India [15], Iran [23], Czech Republic [24], Netherlands [22], Scotland [21], Sweden [19], Canada [18], New Zealand [14], and Australia [12]. The Newcastle–Ottawa scores ranged from 6 to 8 (Table 1). Based on the risk of a bias assessment tool, the included studies had the highest risk associated with patient selection due to the retrospective nature of their designs (Supplement Figures S1 and S2).

### 3.3. Analysis of Pooled Data

We conducted a thorough analysis and comparison of the OS rates for 2-, 3-, and 5-year periods between TL and CRT. Additionally, we examined the OS rates along with the 5-year DSS, specifically focusing on TL and RT in T3 laryngeal cancers.

The pooled results of 12 studies [12,13,14,15,16,17,18,20,21,23,25,26] with separate information on CRT and RT were calculated for 2-year OS as TL = 73%, CRT = 74.7%, RT = 57.9%; 3-year OS TL = 64.3%, CRT = 62.9%, RT = 52.4%; and 5-year OS TL = 54.2%, CRT = 52.7%, RT = 40.8%. Additionally, the pooled 5-year DSS rates for TL and RT were 70.1% and 50.7%, respectively. The data from four studies [12,13,14,15,17,18,21] that did not provide separate information for CRT and RT only were not included in this meta-analysis. 

### 3.4. 2-Year Overall Survival

#### 3.4.1. TL vs. CRT

**Figure 2:**
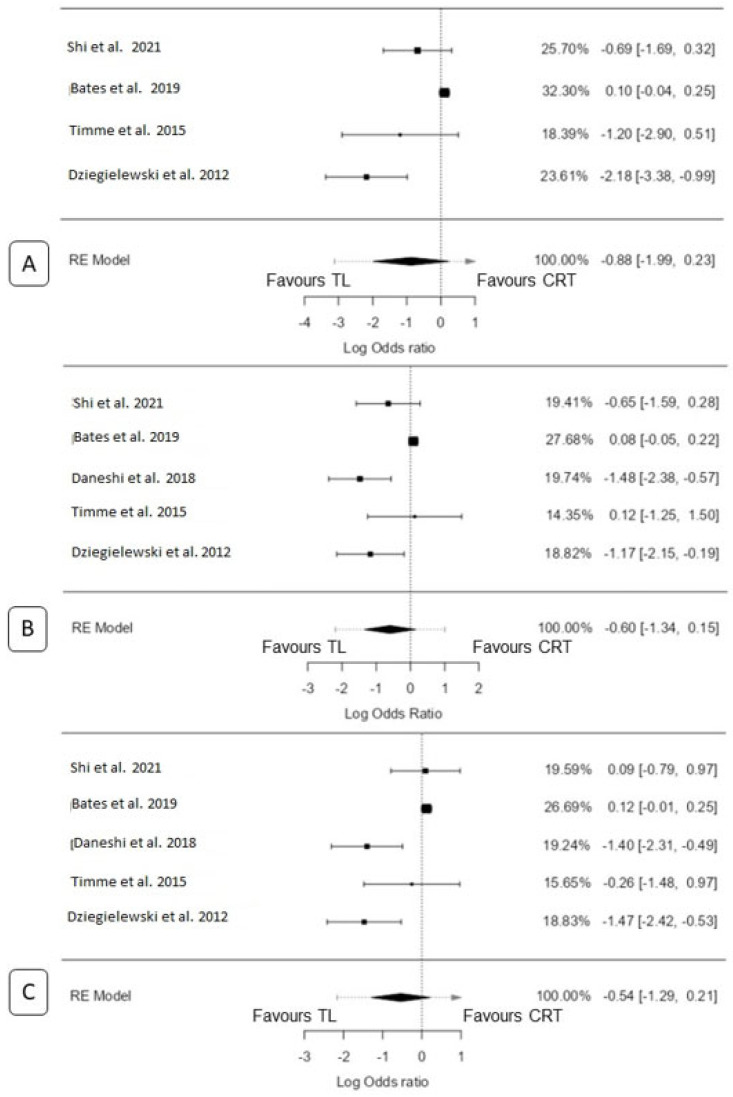
Forest plot for total laryngectomy vs. chemoradiation: (**A**) 2 y OS, (**B**) 3 y OS, and (**C**) 5 y OS [18,20,23,25,26].

A total of four studies [18,20,25,26] were included in the analysis. The observed log odds ratios ranged from −2.18 to 0.10, with the majority of estimates favouring TL (75%). The estimated average log odds ratio based on the random-effects model was −0.88 (95% CI: −1.99 to 0.23). Therefore, the mean outcome did not differ significantly from zero (z = −1.55, *p* = 0.12). According to the Q-test, the true outcomes were heterogeneous (Q(3) = 18.05, *p* = 0.0004, tau^2^ = 0.99, I^2^ = 83.38%). A 95% prediction interval for the true outcomes was −3.12 to 1.37. Although the average outcome was estimated to favour the TL, in three studies [18,20,26], the true outcome favoured CRT. Maximum weightage (32.3%) for this analysis was from Bates et al. [25]. An examination of the studentized residuals revealed that none of the studies had a value larger than ±2.49, indicating no outliers in the context of this model. According to the Cook’s distances, none of the studies could be considered overly influential. The regression test indicated funnel plot asymmetry (*p* = 0.038), but not the rank correlation test (*p* = 0.75) (Figure 2A).

#### 3.4.2. TL vs. RT only

**Figure 3:**
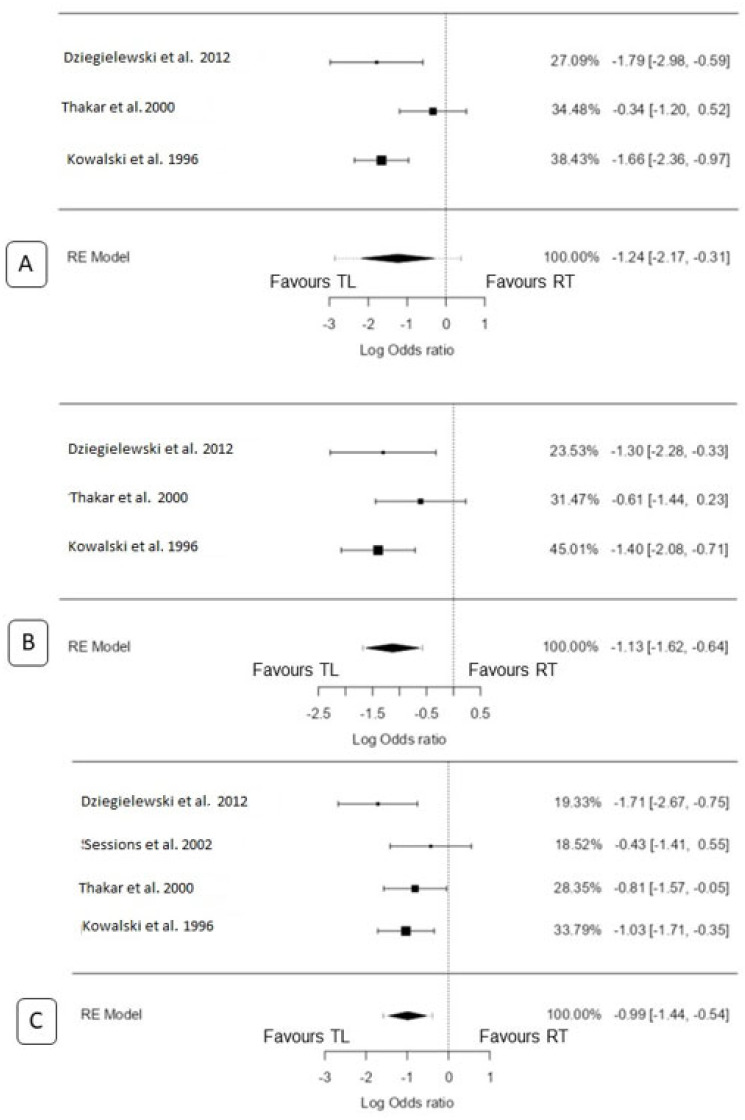
Forest plot for total laryngectomy vs. radiation only: (**A**) 2 y OS, (**B**) 3 y OS, and (**C**) 5 y OS [13,15,17,18].

A total of three studies [13,15,18] were included in the analysis. The observed log odds ratios ranged from −1.78 to −0.34, with all estimates favouring TL (100%). Using the random-effects model, the estimated average log odds ratio was −1.2383 (95% CI: −2.17 to −0.31). Therefore, the mean outcome significantly differed from zero (z = −2.61, *p* = 0.009). Based on the Q-test, there was evidence of heterogeneity in the true outcomes (Q(2) = 6.46, *p* = 0.04, tau^2^ = 0.46, I^2^ = 69.01%). The 95% prediction interval for the true outcomes was −2.86 to 0.38. Maximum weightage (38.4%) for this analysis was from Kowalski et al. [13]. An examination of the studentized residuals revealed that Thakar et al. [15] had a value larger than ±2.39 and may be a potential outlier in the context of this model. However, according to the Cook’s distances, none of the studies could be considered overly influential. Neither the rank correlation test nor the regression test indicated any funnel plot asymmetry (*p* = 1.0 and *p* = 0.83, respectively) (Figure 3A).

### 3.5. 3-Year Overall Survival

#### 3.5.1. TL vs. CRT

A total of five studies were included in the analysis [18,20,23,25,26]. The observed log odds ratios ranged from −1.48 to 0.12, with the majority of estimates favouring TL (60%). The estimated mean log odds ratio based on the random-effects model was −0.59 (95% CI: −1.34 to 0.15). Therefore, the mean outcome did not differ significantly from zero (z = −1.57, *p* = 0.11). According to the Q-test, the true outcomes appeared to be heterogeneous (Q(4) = 18.9642, *p* = 0.0008, tau^2^ = 0.51, I^2^ = 78.91%). A 95% prediction interval for the true outcomes was given by −2.19 to 0.99. Hence, although the average outcome was estimated to favour TL, in three studies [18,23,26], the true outcome favoured CRT. Maximum weightage (27.7%) for this analysis was from Bates et al. [25]. An examination of the studentized residuals revealed that none of the studies had a value larger than ±2.58, indicating no outliers in the context of this model. According to the Cook’s distances, none of the studies could be considered overly influential. Neither the rank correlation test nor the regression test indicated any funnel plot asymmetry (*p* = 1.0 and *p* = 0.36, respectively) (Figure 2B).

#### 3.5.2. TL vs. RT Only

A total of three studies were included in the analysis [13,15,18]. The observed log odds ratios ranged from −1.40 to −0.61, with all estimates favouring TL (100%). Using the random-effects model, the estimated average log odds ratio was −1.13 (95% CI: −1.62 to −0.64). Therefore, the mean outcome differed significantly from zero (z = −4.50, *p* < 0.0001). According to the Q-test, there was no significant amount of heterogeneity in the true outcomes (Q(2) = 2.19, *p* = 0.33, tau^2^ = 0.02, I^2^ = 8.74%). The 95% prediction interval for the true outcomes was −1.68 to −0.57. Hence, even though there may be some heterogeneity, the true outcomes of the studies were generally in the same direction as the estimated average outcome (favouring TL). Maximum weightage (27.7%) for this analysis was from Thakar et al. [15]. An examination of the studentized residuals revealed that none of the studies had a value larger than ±2.39, indicating no outliers in the context of this model. Furthermore, according to the Cook’s distances, none of the studies was considered overly influential. Neither the rank correlation test nor the regression test indicated any funnel plot asymmetry (*p* = 1.00 and *p* = 0.82, respectively) (Figure 3B).

### 3.6. 5-Year Overall Survival

#### 3.6.1. TL vs. CRT

A total of five studies were included in the analysis [18,20,23,25,26]. The observed log odds ratios ranged from −1.47 to 0.12, with the majority of estimates favouring TL (60%). Using the random-effects model, the estimated average log odds ratio was −0.54 (95% CI: −1.29 to 0.21). Therefore, the mean outcome did not significantly differ from zero (z = −1.41, *p* = 0.16). According to the Q-test, there was evidence of heterogeneity in the true outcomes (Q(4) = 21.05, *p* = 0.0003, tau^2^ = 0.54, I^2^ = 80.99%). The 95% prediction interval for the true outcomes was −2.16 to 1.09. This suggests that although the average outcome was estimated to be negative, in three studies [18,20,23], the true outcome in fact favoured CRT. Maximum weightage (26.7%) for this analysis was from Bates et al. [25]. An examination of the studentized residuals revealed that none of the studies had a value larger than ±2.58, indicating no outliers in the context of this model. Additionally, according to the Cook’s distances, none of the studies were considered overly influential. Neither the rank correlation test nor the regression test indicated any funnel plot asymmetry (*p* = 0.23 and *p* = 0.34, respectively) (Figure 2C).

#### 3.6.2. TL vs. RT Only

A total of four studies were included in the analysis [13,15,17,18]. The observed log odds ratios ranged from −1.71 to −0.43, with all estimates favouring TL (100%). Using the random-effects model, the estimated mean log odds ratio was −0.99 (95% CI: −1.44 to −0.54). Therefore, the mean outcome differed significantly from zero (z = −4.27, *p* < 0.0001). According to the Q-test, there was no significant amount of heterogeneity in the true outcomes (Q(3) = 3.64, *p* = 0.30, tau^2^ = 0.04, I^2^ = 17.58%). The 95% prediction interval for the true outcomes was −1.58 to −0.40. Hence, even though there may be some heterogeneity, the true outcomes of the studies were generally in the same direction as the estimated average outcome (favouring TL). Maximum weightage (33.8%) for this analysis was from Kowalski et al. [13]. An examination of the studentized residuals revealed that none of the studies had a value larger than ±2.50, indicating no outliers in the context of this model. Furthermore, according to the Cook’s distances, none of the studies was considered overly influential. Neither the rank correlation test nor the regression test indicated any funnel plot asymmetry (*p* = 0.75 and *p* = 0.92, respectively) (Figure 3C).

### 3.7. 5-Year Disease-Specific Survival

#### TL vs. RT Only

**Figure 4:**
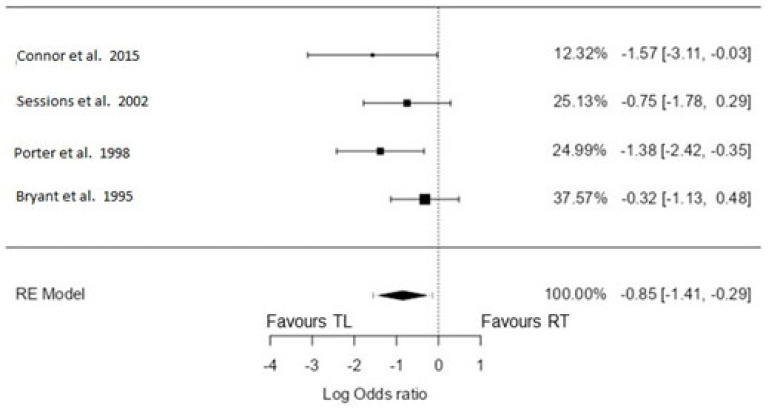
Forest plot for total laryngectomy vs. radiation only: 5-year DSS [12,14,17,21].

**Figure 5:**
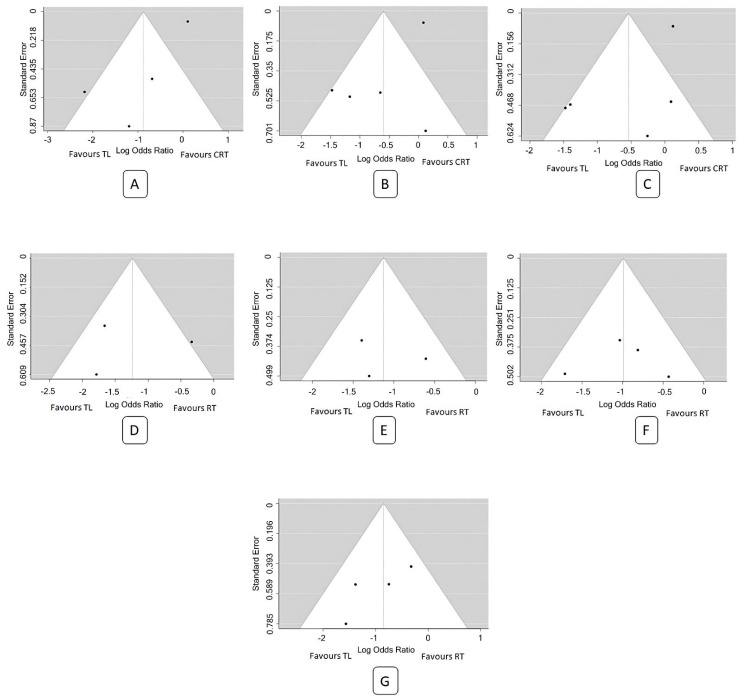
Funnel plot for meta-analysis: (**A**) 2 y OS (TLvsCRT), (**B**) 3 y OS (TLvsCRT), (**C**) 5 y OS (TLvsCRT), (**D**) 2 y OS (TLvsRT), (**E**) 3 y OS (TLvsRT), (**F**) 5 y OS (TLvsRT), and (**G**) 5 y DSS (TLvsRT).

A total of four studies were included in the analysis [12,14,17,21]. The observed log odds ratios ranged from −1.57 to −0.32, with all estimates favouring TL (100%). Using the random-effects model, the estimated average log odds ratio was −0.85 (95% CI: −1.41 to −0.29). Therefore, the mean outcome differed significantly from zero (z = −2.96, *p* = 0.003). According to the Q-test, there was no significant amount of heterogeneity in the true outcomes (Q(3) = 3.50, *p* = 0.32, tau^2^ = 0.05, I^2^ = 14.25%). The 95% prediction interval for the true outcomes was −1.55 to −0.14. Hence, even though there may be some heterogeneity, the true outcomes of the studies were generally in the same direction as the estimated average outcome (favouring TL). Maximum weightage (33.8%) for this analysis was from Bryant et al. [12]. An examination of the studentized residuals revealed that none of the studies had a value larger than ±2.50, indicating no outliers in the context of this model. Furthermore, according to the Cook’s distances, none of the studies was considered overly influential. Neither the rank correlation test nor the regression test indicated any funnel plot asymmetry (*p* = 0.33 and *p* = 0.13, respectively) (Figure 4). The funnel plots are depicted in Figure 5.

## 4. Discussion

Our study is the first to systematically assess OS and DSS exclusively in T3 laryngeal cancers undergoing TL and organ preservation protocols (CRT or RT only). This meta-analysis includes 16 studies from 11 countries, providing a diverse set of patient populations. The statistical analysis conducted is transparent and robust. In the comparison of TL and CRT, most estimates showed that TL had marginally better absolute difference in overall survival outcomes across all time points. However, due to significant heterogeneity among the studies, and the mean outcome did not demonstrate a statistically significant difference. In the comparison of TL and RT only, all estimates significantly favoured TL. 

The decision-making process for T3 laryngeal cancers is complex, requiring a comprehensive understanding of various outcome assessment domains such as survival, function, treatment-associated morbidity, duration, and cost of treatment [39,40]. The choice between surgery and non-surgical protocols, such as organ preservation, requires a thorough understanding of the available evidence. While organ preservation protocols can achieve laryngeal preservation rates, they do not necessarily confer a long-term survival advantage compared to primary TL. Moreover, potential complications and side effects are associated with organ preservation, such as dysphagia, aspiration, RT-related toxicity, and the need to salvage TL. On the other hand, loss of natural voice and permanent tracheostomy can cause serious psychological trauma in operated patients. Thus, patient selection, open communication, and shared decision making are essential in determining the most appropriate treatment approach for individual patients.

The reviews of retrospective reports on non-surgical treatment of locally advanced laryngeal cancer pointed to the experience of the diagnostic and therapeutic team to be of crucial importance for a favourable outcome. However, the landmark Veterans Affairs (VA) prospective randomised study published in 1991 [2] established no difference in 10-year OS between organ preservation and surgical arms [41]. In the VA trial, the subset of laryngeal cancer classified as cT3 accounted for 65% of the cases [2], while in the RTOG 91-11 trial, it constituted 79% of the cases [42].

### 4.1. Survival Outcomes between Total Laryngectomy and Non-Surgical Protocols

The main goals of the non-surgical organ preservation trials were to assess the feasibility of preserving the larynx through non-surgical approaches without compromising the survival outcomes. It is important that although the primary endpoint of these non-surgical organ preservation studies was not focused on survival, none of them has indicated any additional benefit in terms of survival compared to primary TL for cT3 cancer [4,43,44]. The Pignon et al. meta-analysis included three trials (VA, EORTC, and GETTEC), including 602 patients with advanced larynx and hypopharynx carcinomas who underwent TL plus RT or NACT followed by RT. Although there was a negative survival effect of non-surgical treatment on survival (at 5 years: 39% vs. 45% in the TL group), the study showed no significant survival differences in a pooled analysis [45]. In our meta-analysis, the pooled results showed that the 2-year OS rates were 73% for TL, 74.7% for CRT, and 57.9% for RT. For the 3-year OS, the rates were 64.3% for TL, 62.9% for CRT, and 52.4% for RT. The 5-year OS rates were 54.2% for TL, 52.7% for CRT, and 40.8% for RT. Additionally, the pooled 5-year DSS rates were 70.1% for TL and 50.7% for RT. Absolute differences between TL and CRT at all observed time-points were negligible: the average outcome was estimated to favour the TL; in three studies [18,20,26], the true outcome favoured CRT. However, RT showed significantly lower survival outcomes when compared to the other two treatment scenarios.

On meta-analysis, there was a no statistically significant difference in the OS between TL and CRT and there was a statistically significant difference in the OS and DSS between TL and RT alone. It is essential to mention there was a statistically significant heterogeneity among the included studies for OS estimation between TL and CRT and for 2-year OS between TL and RT. Bates et al. [25], analysing the National Cancer Database (NCDB), had the highest weightage in determining the OS between TL and CRT due their large sample size. 

It is important to note that the studies focusing on RT alone for the treatment of T3 laryngeal cancers were published between 1995 and 2015. Even though the study by Connor et al. [21] was published in 2015, it included cases recruited from 1999 to 2010. In their cohort, it was observed that there was a temporal shift in the treatment approach over time. RT alone was primarily employed in patients treated earlier, but a notable change occurred with a shift towards CRT in later years. This finding suggests that the treatment strategy for T3 laryngeal cancers evolved over the time, with a transition from RT alone to the combined CRT approach. Additionally, the RT alone group accounted for only 2.7% of the cases, indicating that the use of RT as the sole treatment for T3 laryngeal cancers is nowadays less commonly used and limited only to inoperable and poor performance cases that are not suitable for RT intensification with the addition of chemotherapy.

Tang et al. conducted a meta-analysis which focused on the comparison of survival outcomes between patients with a mix of advanced laryngeal cancer who underwent non-surgical organ preservation protocols versus TL. The subgroup analysis of T3 tumours revealed that OS was not significantly worse for the non-surgical group compared to primary TL, with a hazard ratio of 0.96. Their meta-analysis included studies from 1995 to 2016. A forest plot for the T3 subgroup and temporal frame for OS was not provided in their study [46].

### 4.2. Organ Preservation with Non-Surgical Protocols

The VA group reported a 64% laryngectomy-free survival at 2 years after three cycles of chemotherapy with cisplatin and 5 fluorouracil followed by definitive RT [2]. The RTOG 91-11 trial established concurrent CRT as the current standard for organ preservation protocols, demonstrating a nearly 54% relative risk reduction in local recurrence compared to RT alone. The CRT arm demonstrated 88% rate of laryngeal preservation compared to 75% in NACT followed by the RT arm, and 70% for the RT arm alone [42]. The GORTEC [3] and RTOG 91-11 [42] studies suggested that adding NACT to organ preservation protocols did not improve control rates. In the present study, we did not assess the impact of NACT on survival or organ preservation.

In our meta-analysis, we came across several articles that examined the rates of organ preservation or laryngectomy-free survival associated with non-surgical protocols. Nguyen-Tan et al. reported a 5-year laryngeal preservation rate of 64% in the CRT arm [16]. Connor et al. found that 69.4% of patients receiving non-surgical organ preservation therapy had a normal voice at 6 months, with 25% having a functional voice. Among those who underwent TL, 83% had a functional voice with a voice prosthesis [21]. Sessions et al. reported a laryngeal preservation rate of 69.5% in their cohort [17]. Timmermans et al. demonstrated a 5-year laryngectomy-free survival of 81% in the RT arm and 86.9% in the CRT arm [22]. It is important to highlight that the funnel plots demonstrate poor outcomes with non-surgical protocols in older studies, indicating that our understanding of the disease biology has improved, along with advancements in non-surgical treatment techniques (Figure 5). In the GORTEC group study, the 5-year and 10-year laryngeal preservation rates were 74% and 58%, respectively [47]. 

### 4.3. Main Reasons for Failures of Non-Surgical Organ Preservation

The VA trial reported that salvage TL was needed in 56% of stage T4 laryngeal cancer patients and in 29% of patients with smaller tumours, suggesting that patients with cartilage involvement may not be suitable candidates for organ preservation [2,48,49]. According to the study of Connor et al., RT may not be effective for cases with a tumour volume exceeding 6 cm^3^ [50]. A recent study by Malik et al. concluded that in patients with T3 laryngeal cancers, a one cm^3^ increase in tumour volume led to significantly worse OS (HR = 1.07) and DFS (HR = 1.11) [51]. In the study by Sharrett et al., the locoregional failure at 2 years post-CRT was found to be 39%. On univariate analysis, the risk of local and regional failures was associated with primary tumour (*p* = 0.05) and nodal volumes (*p* = 0.04) [52]. Hamilton et al. found a tumour volume of >3 cm^3^ to be a significant predictor of locoregional failure [53]. Interestingly, Hanubal et al. reported that a primary tumour volume of more than 7.1 cm^3^ in T3 laryngeal cancers undergoing TL was found to have a worse prognosis [54]. Tangsriwong et al. [55] found that N category, volume of primary, vocal cord involvement, dose of RT, and radiation treatment gap exceeding seven days were identified as factors that significantly influenced laryngeal preservation. Pechacova et al. [56] reported that baseline performance status, weight loss, comorbidities, coexisting anaemia, history of alcohol intake, and marital status were predictors of OS in patients receiving CRT for laryngeal cancer. Sherman et al. identified that patients with T stage tumours (T4), serum albumin (<4 g/dL), alcohol use (≥6 drinks/day), and Karnofsky’s score (<80) had significantly decreased rates of larynx preservation [57].

### 4.4. Morbidity following TL

TL is a well-established surgical procedure for the treatment of laryngeal cancers. In the study by Pantvaidya et al., 65% of their cohort underwent TL as the primary treatment modality for advanced laryngeal (and hypopharyngeal) cancers, including cases with dysfunctional larynx at the tumour diagnosis [58]. Forty two percent of the patients underwent various forms of reconstruction, with the pectoralis major myocutaneous flap being the predominant technique employed. Pharyngocutaneous fistula (PCF) emerged as the most frequently encountered major complication following TL, with reported incidence rates ranging from 2% to 65%. However, in the majority of cases, conservative management, including dressings, antibiotics, and supportive care, proved sufficient to lower the fistula rate, while surgical intervention was required in only a minority (9%) of patients. Overall postoperative mortality stood at 1.7%, with three deaths resulting from complications such as major PCFs, carotid blowouts, and gastrostomy tube induced necrosis. Distant metastases accounted for a substantial proportion (42%) of total recurrences in the analysed patient cohort [58]. Following total laryngectomy, the loss of voice can have a profound impact on patient’s quality of life, causing a considerable decline in overall well-being and leading to significant frustration due to the challenges in effective communication [59].

### 4.5. Morbidity following CRT and RT 

Following organ preservation protocols, it is vital to consider complications such as cartilage necrosis, enteral feeds, dysphagia, voice impairments, and tracheostomy rates. Thus, the laryngeal preservation protocols do not necessarily translate into a better quality of life than TL. In the RTOG 91-11 follow-up analysis, severe late dysphagia after CRT was reported in 26.5% of patients, and 10% required permanent enteral nutrition [60]. In a study by Staton et al., 36% of advanced laryngeal cancer patients undergoing non-surgical organ preservation had persistent tracheostomy tube following therapy [61]. However, these studies are of cohorts of patients treated decades ago. Newer intensity-modulated RT techniques have the potential to reduce laryngeal toxicity, with the recommended dose to avoid laryngeal oedema being between 43 to 45 Gy [62]; however, with the required tumoricidal dose of 66–70 Gy to the larynx, compliance with these dose limits is not possible [63,64]. 

### 4.6. Salvage Laryngectomy

The RTOG 91-11 study indicated that salvage TL was required in 16% of patients in the CRT arm, 28% in the NACT followed by RT arm, and 31% in the RT-only arm [42]. However, the 2-year OS rates after salvage TL in this study ranged from 69% to 76% across all treatment arms [49,65]. Shoushtari et al. compared the survival outcomes between primary and salvage laryngectomy at 2-, 3-, and 5-years post-surgery. They found that the 2-year OS was 79% for primary and 38% for salvage laryngectomy; at 3 years, 70% and 38%; and at 5 years, 64% and 38%, respectively [66]. This highlights a significant reduction in OS rates after salvage TL compared to primary TL. In a meta-analysis by Hasan et al., the overall rate of complications after salvage TL was 67.5% and the rate of PCF was 28.9% [67].

### 4.7. Pointers to Choose the Appropriate Treatment Modality

It is important to consider that patients still consider TL mutilating and this surgery can lead to significant psychological trauma [68]. In a recent study by Laccourreye et al., the main reasons to refuse TL were fear of surgery, permanent tracheostoma, and loss of phonation [69]. Before considering a TL, an open-ended dialogue between surgeon, patient, her/his family members, caregivers, and speech-language pathologist regarding the treatment outcomes is crucial. The reason for the tumour being classified as T3 (e.g., cord fixation, paraglottic space involvement, pre-epiglottic space extension, and cartilage erosion), should be documented, as it may influence treatment as well as functional outcomes (even though specific studies on such different prognosticators so far are still lacking). Clinicians may consider employing definitive CRT strategies for patients diagnosed with T3 tumours, provided that there are sufficient resources available for treatment administration, surveillance, and surgical salvage. It is essential for clinicians to not only consider the results from large prospective randomized trials but also consider the impact of geographical, cultural, and socioeconomic factors when making treatment decisions. 

Therefore, selection for either CRT or laryngectomy should depend not only on T classification and consider not only expected oncologic outcomes, but also the expected functional outcome. In the case of an already “a-functional” larynx with an insufficient airway necessitating tracheostomy due to fixation or swallowing problems including aspiration, laryngectomy may be the better option as the chance of functional recovery after larynx preserving treatment will often be disappointing in these cases. Multidisciplinary board members should facilitate informed decision making by the patient by providing comprehensive information about all potential issues associated with organ preservation and TL. The use of decision aids for this process of shared decision making may be helpful [6].

### 4.8. Limitations of Meta-Analysis

All the studies included in the present meta-analysis are retrospective, and patients in the organ preservation group received different regimes of CRT or RT. Significant heterogeneity exists in the extracted data, and some of the survival data were extrapolated from published Kaplan–Meier graphs. Furthermore, information on the tumour volume and its extent inside the larynx were not available from the included studies: thus, further subset analyses based on tumour volume and subsite involvement were not possible. Additionally, many studies did not provide data on patients requiring tracheostomy in the organ preservation group, prior laryngeal function, performance status, and comorbidities.

## 5. Conclusions

For patients with T3 laryngeal cancer, our meta-analysis demonstrates that TL and CRT have similar OS. Instead, the use of RT only is not recommended and should be limited to the patients not suitable for other more aggressive treatment options. However, there is substantial heterogeneity among the included studies. These findings highlight the importance of individualised treatment decisions, considering patient preferences and factors such as functional outcomes and quality of life. Further research, including prospective studies with appropriate sample sizes and standardised outcome measures, is warranted to validate these findings and provide more robust evidence for treatment decision making in cT3 laryngeal cancers.

## Figures and Tables

**Table 1:** Overview of included studies.

	Author	Country	Year	S	NS	*n* (cT3)	TL	OP	2-Year OS		3-Year OS		5-Year OS		5-Year DSS		Type of Non-Surgical Organ Preservation
									TL *	OP *	TL *	OP *	TL *	OP *	TL *	OP *	
1	Bryant et al. [12]	Australia	1995	R	7	97	42	55							18	28	RT alone
2	Kowalski et al. [13]	Brazil	1996	R	7	221	176	45	39	27	51	28	69	29			RT alone
3	Porter et al. [14]	NZ	1998	R	7	71	46	25							16	17	RT alone
4	Thakar et al. [15]	India	2000	R	7	119	65	54	13	14	13	17	18	25			RT alone
5	Nguyen-Tan et al. [16]	USA	2001	R	7	83	70	13									CRT
6	Sessions et al. [17]	USA	2002	R	7	65	36	29					16	16	10	13	RT alone
7	Dziegielewski et al. [18]	Canada	2012	R	7	126	35	91	4	34	8	46	11	63			Both CRT and RT, separate data available
8	Karlsson et al. [19]	Sweden	2014	R	7	71	17	54									CRT and RT, separately not specified in data
9	Timme et al. [20]	USA	2015	R	7	44	19	25	2	7	5	6	11	16			CRT
10	Connor et al. [21]	Scotland	2015	R	7	106	30	76							2	21	Both CRT and RT, separate data available
11	Timmermans et al. [22]	Netherlands	2016	R	7	1922	324	1598	109	510	131	661	180	973			CRT and RT, separately not specified in data
12	Daneshi et al. [23]	Iran	2018	R	7	151	127	24			27	13	37	15			CRT
13	Čoček et al. [24]	Czech Rep.	2018	R	7	70	45	25	8	6	11	8	17	13			CRT and RT, separately not specified in data
14	Bates et al. [25]	USA	2019	R	7	7569	1044	6525	282	1631	407	2414	522	3067			CRT
15	Shi et al. [26]	USA	2021	R	7	88	32	56	7	20	9	24	19	32			CRT
16	Lee et al. [27]	USA	2023	R	7	137	41	96	16	19	20	32	26	51			CRT and RT, not specified in data

All the included studies were level 3 evidence as per the Oxford Centre for Based Medicine (OCEBM) criteria. S—Study type, R—Retrospective, NS—Newcastle–Ottawa Scale, NZ—New Zealand, OS—Overall survival, DSS—Disease-specific survival, TL—Total laryngectomy, OP—Organ preservation, *—Events. CRT—Chemoradiation. RT—Radiation alone.

## Supplementary Materials

The following supporting information can be downloaded at https://www.mdpi.com/article/10.3390/biomedicines11082128/s1. Figure S1: Risk of bias summary; Figure S2: Risk of bias graph.
