# Supplementary material for: Survival Outcomes in T3 Laryngeal Cancers: Primary Total Laryngectomy vs. Concurrent Chemoradiation or Radiation Therapy—A Meta-Analysis
Source: Biomedicines. 2023 Jul 28;11(8):2128. doi: 10.3390/biomedicines11082128 (PMC10452463; doi:10.3390/biomedicines11082128)
Supplement: Supplementary file 1 [file biomedicines-11-02128-s001.zip › biomedicines-2515375-supplementary.pdf]

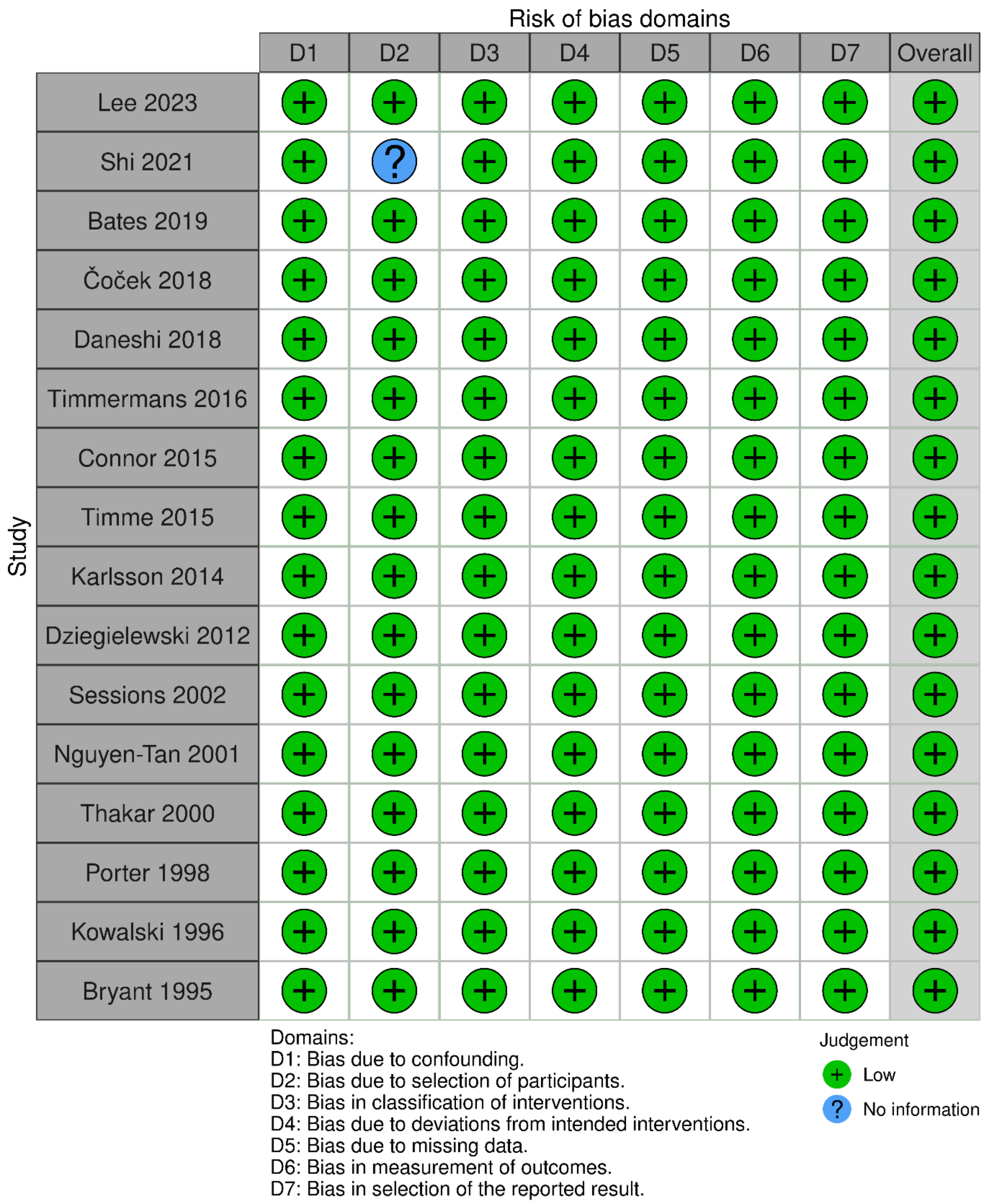

Figure S1: Risk of bias summary

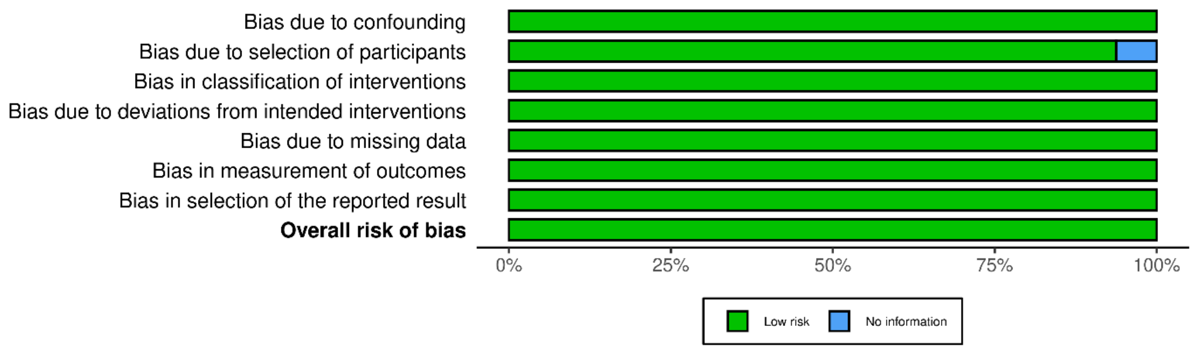

Figure S2: Risk of bias graph
